# MiT/TFE Family Renal Cell Carcinoma

**DOI:** 10.3390/genes14010151

**Published:** 2023-01-05

**Authors:** Jinglong Tang, Masaya Baba

**Affiliations:** Laboratory of Cancer Metabolism, International Research Center for Medical Sciences (IRCMS), Kumamoto University, Kumamoto 860-0811, Japan

**Keywords:** MiT/TFE family, RCC, MITF, TFE3, TFEB, SUMO

## Abstract

The microphthalmia-associated transcription factor/transcription factor E (MiT/TFE) family of transcription factors are evolutionarily conserved, basic helix–loop–helix leucine zipper (bHLH-Zip) transcription factors, consisting of MITF, TFEB, TFE3, and TFEC. MiT/TFE proteins, with the exception of TFEC, are involved in the development of renal cell carcinoma (RCC). Most of the MiT/TFE transcription factor alterations seen in sporadic RCC cases of MiT family translocation renal cell carcinoma (tRCC) are chimeric proteins generated by chromosomal rearrangements. These chimeric MiT/TFE proteins retain the bHLH-Zip structures and act as oncogenic transcription factors. The germline variant of *MITF* p.E318K has been reported as a risk factor for RCC. E 318 is present at the SUMOylation consensus site of MITF. The p.E318K variant abrogates SUMOylation on K 316, which results in alteration of MITF transcriptional activity. Only a few cases of *MITF* p.E318K RCC have been reported, and their clinical features have not yet been fully described. It would be important for clinicians to recognize *MITF* p.E318K RCC and consider *MITF* germline testing for undiagnosed familial RCC cases. This review outlines the involvement of the MiT/TFE transcription factors in RCC, both in sporadic and hereditary cases. Further elucidation of the molecular function of the MiT/TFE family is necessary for better diagnosis and treatment of these rare diseases.

## 1. The Microphthalmia/Transcription Factor E (MiT/TFE) Family

The microphthalmia-associated transcription factor/transcription factor E (MiT/TFE) family consists of MITF, TFEB, TFE3, and TFEC. All of the proteins have a bHLH-Zip (basic helix–loop–helix leucine zipper) structure, which allows them to form homodimers or heterodimers with each other and bind to the regulatory elements of target genes [1,2,3,4]. The binding consensus sequences recognized by the MiT/TFE proteins are known as the E-box motif (CACGTG) and M-box motif (TCATGTG) [5,6,7,8]. The MiT/TFE proteins are the master regulators of lysosomal biogenesis and autophagy [9]. In addition, their fundamental roles in many cellular processes including proliferation, differentiation, survival, senescence, invasion, metabolism, organelle biogenesis, and stress responses are emerging [1,2,3].

## 2. MiT Family Translocation Renal Cell Carcinoma (tRCC)

**Figure 1:**
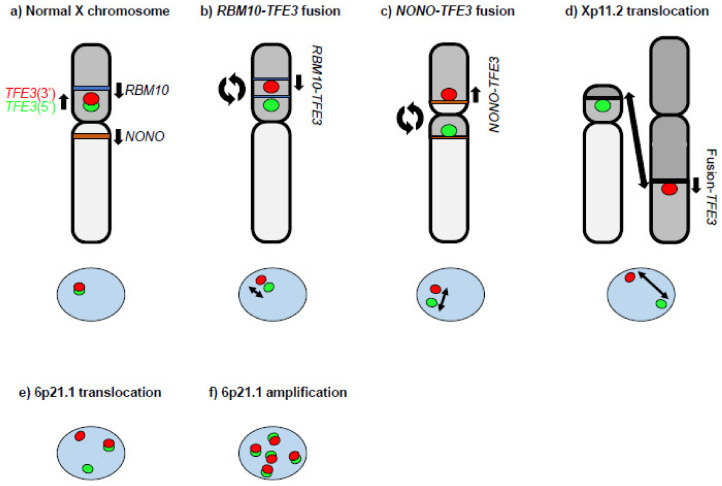
A diagram of *TFE3* gene break-apart FISH. (**a**) The *TFE3* gene is located at chromosome Xp11.2. The 5′ FISH probe for *TFE3* is green. The 3′ FISH probe for *TFE3* is red. A fusion candidate gene, *RBM10* (blue bar), is located at chromosome Xp11.23 on the telomere side of *TFE3*. Another fusion candidate gene, *NONO* (brown bar), is located at chromosome Xq13.1. The *TFE3* gene break-apart FISH demonstrates co-localization of the green and red probes. (**b**) An intra Xp (paracentric) inversion inv(X)(p11.2;p11.23) causes the *RBM10–TFE3* fusion gene. The *TFE3* gene break-apart FISH demonstrates subtle split red and green signals. Two blue bars indicate separated *RBM10*. The length of the two-arrowhead line indicates the relative distance between the FISH signals. (**c**) A pericentric X chromosome inversion, inv(X)(p11.2;q13.1), causes the *NONO–TFE3* fusion gene. The *TFE3* gene break-apart FISH demonstrates slight split red and green signals. The two brown bars indicate separated *NONO*. The length of the two-arrowhead line indicates the relative distance between the FISH signals. (**d**) Xp11.2 translocation may occur with another chromosome. The *TFE3* gene break-apart FISH demonstrates clearly separated green and red signals. The length of the two-arrowhead line indicates the relative distance between the FISH signals. (**e**) The *TFEB* gene is located at chromosome 6p21.1. The 5′ FISH probe for *TFEB* is green. The 3′ FISH probe for *TFEB* is red. 6p21.1 translocated RCC demonstrates clearly separated green and red signals as well as co-localized green and red signals by *TFEB* gene break-apart FISH. (**f**) 6p21.1 amplified RCC demonstrates amplification of co-localized green and red signals by *TFEB* gene break-apart FISH.

**Figure 2:**
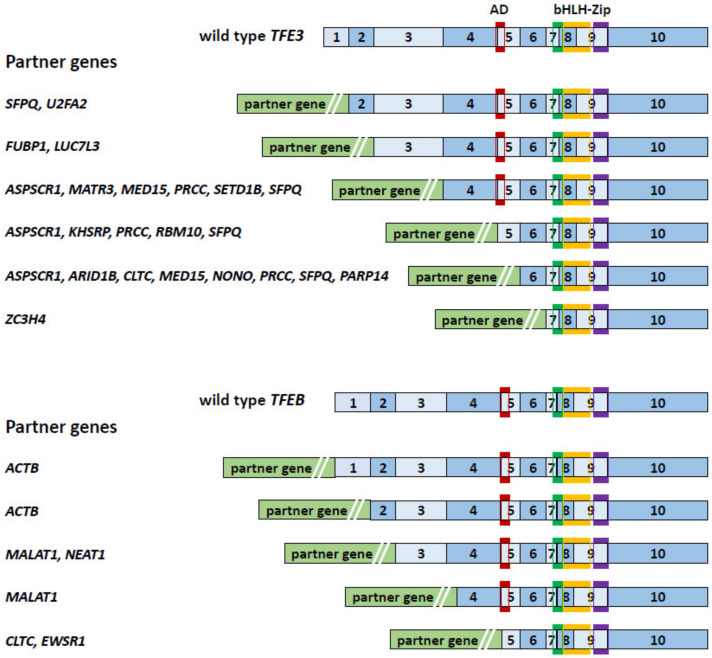
Structures of *TFE3* fusion and *TFEB* fusion genes. The structures of *TFE3* fusions and *TFEB* fusions found in *TFE3*-rearranged RCC and *TFEB*-altered RCC are shown. Wild-type *TFE3* and *TFEB* have 10 exons. The fusion partner genes are listed. All of the fusion genes retain coding exons for the bHLH-Zip domain. AD: activation domain; bHLH-Zip: basic helix–loop–helix leucine zipper.

MiT family translocation renal cell carcinoma (tRCC) is a sporadic RCC characterized by fusion genes involving the MiT/TFE family genes, *MITF*, *TFEB*, and *TFE3* and defined as an MiT family translocation RCC in the 2016 WHO classification [10,11]. In the 2022 WHO classification, tRCC is divided into *TFE3*-rearranged RCC and *TFEB*-altered RCC as Moleculary-defined RCCs [12]. tRCC is a rare disease that accounts for approximately 1–5% of sporadic RCC in adults [13,14,15,16,17], developing more often in women than in men, and is much more commonly seen in pediatric RCC cases (approximately 40% (range 20–75%)) [13,18,19,20,21]. tRCC tends to be in an advanced stage at onset with a more aggressive presentation than other sporadic RCCs, and molecular targeted therapy for advanced cases has not yet been established [10,22]. The most distinctive histopathological features of tRCC are clear-cell papillary, displaying a papillary structure consisting of clear cells. However, recent studies have shown that tRCCs are morphologically heterogenous and can include papillary, tubular, acinar, and even cystic architecture [10,17,23,24]. In fact, some cases are indistinguishable from clear-cell (cc)RCC or papillary (p)RCC by H&E staining alone [16]. Therefore, it is expected that the actual number of tRCC cases may be higher than the number currently diagnosed. Definitive diagnosis requires TFE3/TFEB/MITF immunohistochemistry and FISH [25,26,27], which are not routinely performed in many hospitals. Hence, the immunohistochemistry of surrogate markers such as cathepsin K, Melan A, and GPNMB should be considered for the initial diagnosis of RCC [18,24,28]. Chimeric MiT/TFE proteins are generated by chromosomal rearrangements. Bakouny et al. have reported that among 88 fusion-defined tRCC cases, most fusion genes (88.6%) involved *TFE3*. On the other hand, *TFEB* fusions were reported in only 9.1% and *MITF* fusions in only 2.3% of these cases [16]. A single center study of the largest *TFE3*-rearranged RCC cohort published to date identified 57 *TFE3* fusion genes in 4581 RCCs [17]. Of the 57 cases, 26.3% were *SFPQ-TFE3* fusions and 22.8% were *ASPSCR1-TFE3* fusions. Of note, X chromosomal inversion was noted in 21% (12/57) of cases, which consisted of *NONO-TFE3* (14%) and *RBM10-TFE3* (7%). These X chromosomal inversion cases can be misdiagnosed as non-*TFE3*-rearranged RCC because of the narrow interval between split signals seen with *TFE3* gene break-apart FISH, the gold standard diagnostic test for *TFE3*-rearranged RCC [29,30]. (Figure 1a–d) *TFEB*-altered RCC includes 6p21.1 translocated RCC and 6p21.1 amplified RCC, which demonstrate distinct signals seen with *TFEB* gene break-apart FISH (Figure 1e,f) [31]. All MiT/TFE fusion genes identified to date retain the bHLH-Zip structure (Figure 2) [16,17,22,32,33,34,35,36], suggesting that these MiT/TFE fusion genes function as oncogenic transcription factors. Indeed, overexpression of TFE3 fusion, PRCC–TFE3, in mouse kidneys was shown to cause RCC with aberrant expression of MiT/TFE target genes [28]. There are few recurrent genomic alterations in tRCC other than MiT/TFE gene rearrangement and 9p21.3 deletion [16]. Several genomic alterations, such as *ASPSCR1-TFE3, LUC7L3-TFE3*, and 22q deletion, correlate with poor prognosis [17,18]. Currently, there is no established standard therapy for advanced tRCC [10,22]. However, recent studies suggest that immunotherapy may be effective for advanced tRCC [16,17].

## 3. *MITF* p.E318K RCC

*MITF* is well known as a key molecule in melanocyte biology [1] as well as functioning as an oncogene that is amplified and mutated in sporadic melanomas [37,38]. *MITF* is also involved in the aforementioned tRCC, although at a much lower frequency [16]. These observations provide strong evidence of a carcinogenic role for abnormal MITF activation.

The *MITF* p.E318K germline variant was reported in 2011 in familial and sporadic melanoma cases [39] and in patients with melanoma and RCC concurrently [40]. Carriers of the p.E318K variant (Mi-E318K) exhibited a 14-fold higher risk than controls for developing melanoma and RCC [40]. Although the *MITF* p.E318K variant did not co-segregate in all melanoma cases in the family, linkage analysis in 31 families demonstrated *MITF* p.E318K as a possible intermediate risk variant [39]. Regarding the incidence of germline *MITF* p.E318K variants in RCC, Shuch and colleagues reported that 0.7% (9 of 1235) of RCC patients who underwent a renal panel test for hereditary kidney-cancer-causing genes (RenalNext) were found to have germline *MITF* p.E318K variants [41]. The age distribution of the tested population was significantly younger than the U.S. kidney cancer population (46.2 ± 13.7 vs. 63.2 ± 13.3), suggesting that the panel population may be a high-risk group for hereditary kidney cancer. However, the identification of germline *MITF* p.E318K variants in 0.7% of the panel-tested RCCs was a much higher percentage than expected. In fact, the positive mutation rates for *FLCN*, *FH*, *SDHB*, and *MET* were 1.8%, 1.3%, 0.6%, and 0.2%, respectively, of the RCCs tested by the panel [41]. These data suggest that *MITF* p.E318K RCCs might be under-diagnosed. The histology of *MITF* p.E318K RCC is variable. Among the nine *MITF* p.E318K RCC cases reported by the Shuch group, five were clear-cell RCC, two were papillary RCC, and two were unspecified [41]. Recently, a case of bilateral multifocal type 1 papillary RCC with a *MITF* p.E318K germline variant was reported [42]. The germline variant in this case was also detected by genetic testing using a panel of 18 RCC susceptibility genes and represents the only reported case of bilateral multifocal RCC associated with the germline *MITF* p.E318K variant. Interestingly, all of the tumors examined in this case showed amplification of chromosomes 7 and 17, which are known features of sporadic type 1 papillary RCC [42].

**Figure 3:**
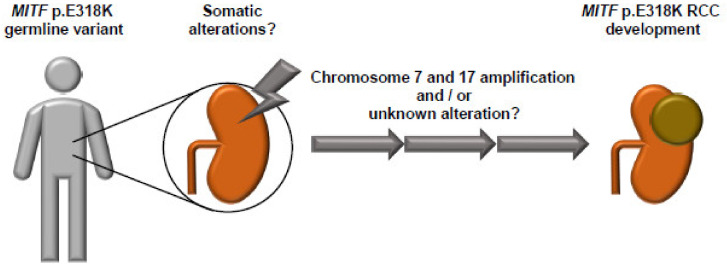
Hypothetical model of *MITF* p.E318K RCC development. The *MITF* p.E318K germline variant is a mild susceptibility allele. In addition to the germline variant, somatic alterations such as chromosome 7 and 17 amplification, unknown gene mutations, or epigenetic alteration might be required for *MITF* p.E318K RCC development.

Glutamic acid (E) residue 318 is located at the small-ubiquitin-like modifier (SUMO) consensus site (YKXE) of MITF. Lysine (K) 316 is the major SUMO acceptor site for MITF. Substitution of K for E at codon 318 significantly impairs SUMO conjugation to MITF on K 316 [43,44]. In general, protein SUMOylation alters protein stability, localization, and protein–protein interactions [45]. Mutations of SUMOylated lysine do not affect MITF dimerization, DNA binding, stability, or nuclear localization [43,44]. SUMOylation of MITF on K316 may regulate the transcriptional activity and target specificity of MITF by an unknown molecular mechanism [46]. The in vivo significance of the *MITF* p.E318K variant was investigated by generating *MITF* p.E318K knock-in mice [47]. *MITF* E318K mice are slightly hypopigmented but show no signs of nevus or melanoma development within 24 months of birth. Although this study did not specifically analyze the development of kidney tumors, the normal lifespan of the *MITF* E318K mice suggests a mild or no kidney phenotype. MITF E318K enhances BRaf-V600E-induced nevus formation by inhibiting BRaf-V600E-induced senescence [47]. This evidence suggests that *MITF* p.E318K is not a strong oncogene, but rather a mild susceptibility allele. Some additional somatic gene alterations might be required for *MITF* p.E318K RCC development. (Figure 3) This idea is supported by the report of bilateral multifocal type 1 papillary RCC displaying amplification of chromosomes 7 and 17 in a patient with the germline *MITF* p.E318K variant [42]. To date, no other genome-wide study of *MITF* p.E318K RCC has been published, which will be necessary to identify additional genomic or epigenomic alterations and elucidate the molecular mechanisms of *MITF* p.E318K RCC pathogenesis. Clinical manifestations and the prognosis of patients with *MITF* p.E318K RCC are largely unknown because of the paucity of reports. In addition, there is no established standard therapy for advanced *MITF* p.E318K RCC [48].

## 4. Conclusions

Both MiT family translocation renal cell carcinoma (tRCC) and *MITF* p.E318K RCC are rare and not widely recognized by clinicians. In the future, it will be necessary to increase awareness of these MiT/TFE family RCCs and develop biomarkers to facilitate diagnosis. Further case series are expected to clarify the clinical pathophysiology and provide the basis for effective treatment of these MiT/TFE family RCCs. In addition, the precise molecular mechanisms for the development of MiT/TFE family RCCs are not yet fully clarified. Aberrant MiT/TFE family transcriptional activation is also seen in RCC that develops in the setting of Birt–Hogg–Dubé syndrome and tuberous sclerosis complex (TSC) [49,50]. Further elucidation of the molecular function of the MiT/TFE family in RCC will lead to a better understanding of BHD-and TSC-associated RCC as well as MiT/TFE family RCC.

## References

[1] Goding C.R., Arnheiter H. (2019). MITF-the first 25 years. Genes Dev..

[2] Napolitano G., Ballabio A. (2016). TFEB at a glance. J. Cell Sci..

[3] La Spina M., Contreras P.S., Rissone A., Meena N.K., Jeong E., Martina J.A. (2020). MiT/TFE Family of Transcription Factors: An Evolutionary Perspective. Front. Cell Dev. Biol..

[4] Beckmann H., Kadesch T. (1991). The leucine zipper of TFE3 dictates helix-loop-helix dimerization specificity. Genes Dev..

[5] Hemesath T.J., Steingrimsson E., McGill G., Hansen M.J., Vaught J., Hodgkinson C.A., Arnheiter H., Copeland N.G., Jenkins N.A., Fisher D.E. (1994). Microphthalmia, a critical factor in melanocyte development, defines a discrete transcription factor family. Genes Dev..

[6] Strub T., Giuliano S., Ye T., Bonet C., Keime C., Kobi D., Le Gras S., Cormont M., Ballotti R., Bertolotto C. (2011). Essential role of microphthalmia transcription factor for DNA replication, mitosis and genomic stability in melanoma. Oncogene.

[7] Aksan I., Goding C.R. (1998). Targeting the microphthalmia basic helix-loop-helix-leucine zipper transcription factor to a subset of E-box elements in vitro and in vivo. Mol. Cell. Biol..

[8] Pogenberg V., Ogmundsdottir M.H., Bergsteinsdottir K., Schepsky A., Phung B., Deineko V., Milewski M., Steingrimsson E., Wilmanns M. (2012). Restricted leucine zipper dimerization and specificity of DNA recognition of the melanocyte master regulator MITF. Genes Dev..

[9] Settembre C., Di Malta C., Polito V.A., Garcia Arencibia M., Vetrini F., Erdin S., Erdin S.U., Huynh T., Medina D., Colella P. (2011). TFEB links autophagy to lysosomal biogenesis. Science.

[10] Argani P. (2022). Translocation carcinomas of the kidney. Genes Chromosomes Cancer.

[11] Moch H., Cubilla A.L., Humphrey P.A., Reuter V.E., Ulbright T.M. (2016). The 2016 WHO Classification of Tumours of the Urinary System and Male Genital Organs-Part A: Renal, Penile, and Testicular Tumours. Eur. Urol..

[12] Moch H., Amin M.B., Berney D.M., Comperat E.M., Gill A.J., Hartmann A., Menon S., Raspollini M.R., Rubin M.A., Srigley J.R. (2022). The 2022 World Health Organization Classification of Tumours of the Urinary System and Male Genital Organs-Part A: Renal, Penile, and Testicular Tumours. Eur. Urol..

[13] Sukov W.R., Hodge J.C., Lohse C.M., Leibovich B.C., Thompson R.H., Pearce K.E., Wiktor A.E., Cheville J.C. (2012). TFE3 rearrangements in adult renal cell carcinoma: Clinical and pathologic features with outcome in a large series of consecutively treated patients. Am. J. Surg. Pathol..

[14] Komai Y., Fujiwara M., Fujii Y., Mukai H., Yonese J., Kawakami S., Yamamoto S., Migita T., Ishikawa Y., Kurata M. (2009). Adult Xp11 translocation renal cell carcinoma diagnosed by cytogenetics and immunohistochemistry. Clin. Cancer Res..

[15] Zhong M., De Angelo P., Osborne L., Paniz-Mondolfi A.E., Geller M., Yang Y., Linehan W.M., Merino M.J., Cordon-Cardo C., Cai D. (2012). Translocation renal cell carcinomas in adults: A single-institution experience. Am. J. Surg. Pathol..

[16] Bakouny Z., Sadagopan A., Ravi P., Metaferia N.Y., Li J., AbuHammad S., Tang S., Denize T., Garner E.R., Gao X. (2022). Integrative clinical and molecular characterization of translocation renal cell carcinoma. Cell Rep..

[17] Sun G., Chen J., Liang J., Yin X., Zhang M., Yao J., He N., Armstrong C.M., Zheng L., Zhang X. (2021). Integrated exome and RNA sequencing of TFE3-translocation renal cell carcinoma. Nat. Commun..

[18] Qu Y., Wu X., Anwaier A., Feng J., Xu W., Pei X., Zhu Y., Liu Y., Bai L., Yang G. (2022). Proteogenomic characterization of MiT family translocation renal cell carcinoma. Nat. Commun..

[19] Van der Beek J.N., Hol J.A., Coulomb-l’Hermine A., Graf N., van Tinteren H., Pritchard-Jones K., Houwing M.E., de Krijger R.R., Vujanic G.M., Dzhuma K. (2021). Characteristics and outcome of pediatric renal cell carcinoma patients registered in the International Society of Pediatric Oncology (SIOP) 93-01, 2001 and UK-IMPORT database: A report of the SIOP-Renal Tumor Study Group. Int. J. Cancer.

[20] Van der Beek J.N., Geller J.I., de Krijger R.R., Graf N., Pritchard-Jones K., Drost J., Verschuur A.C., Murphy D., Ray S., Spreafico F. (2020). Characteristics and Outcome of Children with Renal Cell Carcinoma: A Narrative Review. Cancers.

[21] Argani P. (2015). MiT family translocation renal cell carcinoma. Semin. Diagn. Pathol..

[22] Kauffman E.C., Ricketts C.J., Rais-Bahrami S., Yang Y., Merino M.J., Bottaro D.P., Srinivasan R., Linehan W.M. (2014). Molecular genetics and cellular features of TFE3 and TFEB fusion kidney cancers. Nat. Rev. Urol..

[23] Kuroda N., Mikami S., Pan C.C., Cohen R.J., Hes O., Michal M., Nagashima Y., Tanaka Y., Inoue K., Shuin T. (2012). Review of renal carcinoma associated with Xp11.2 translocations/TFE3 gene fusions with focus on pathobiological aspect. Histol. Histopathol..

[24] Caliò A., Brunelli M., Segala D., Pedron S., Remo A., Ammendola S., Munari E., Pierconti F., Mosca A., Bollito E. (2020). Comprehensive analysis of 34 MiT family translocation renal cell carcinomas and review of the literature: Investigating prognostic markers and therapy targets. Pathology.

[25] Zhong M., De Angelo P., Osborne L., Keane-Tarchichi M., Goldfischer M., Edelmann L., Yang Y., Linehan W.M., Merino M.J., Aisner S. (2010). Dual-color, break-apart FISH assay on paraffin-embedded tissues as an adjunct to diagnosis of Xp11 translocation renal cell carcinoma and alveolar soft part sarcoma. Am. J. Surg. Pathol..

[26] Argani P., Yonescu R., Morsberger L., Morris K., Netto G.J., Smith N., Gonzalez N., Illei P.B., Ladanyi M., Griffin C.A. (2012). Molecular confirmation of t(6;11)(p21;q12) renal cell carcinoma in archival paraffin-embedded material using a break-apart TFEB FISH assay expands its clinicopathologic spectrum. Am. J. Surg. Pathol..

[27] Skala S.L., Xiao H., Udager A.M., Dhanasekaran S.M., Shukla S., Zhang Y., Landau C., Shao L., Roulston D., Wang L. (2018). Detection of 6 TFEB-amplified renal cell carcinomas and 25 renal cell carcinomas with MITF translocations: Systematic morphologic analysis of 85 cases evaluated by clinical TFE3 and TFEB FISH assays. Mod. Pathol..

[28] Baba M., Furuya M., Motoshima T., Lang M., Funasaki S., Ma W., Sun H.W., Hasumi H., Huang Y., Kato I. (2019). TFE3 Xp11.2 Translocation Renal Cell Carcinoma Mouse Model Reveals Novel Therapeutic Targets and Identifies GPNMB as a Diagnostic Marker for Human Disease. Mol. Cancer Res..

[29] Kato I., Furuya M., Baba M., Kameda Y., Yasuda M., Nishimoto K., Oyama M., Yamasaki T., Ogawa O., Niino H. (2019). RBM10-TFE3 renal cell carcinoma characterised by paracentric inversion with consistent closely split signals in break-apart fluorescence in-situ hybridisation: Study of 10 cases and a literature review. Histopathology.

[30] Liu N., Guo W., Shi Q., Zhuang W., Pu X., Chen S., Qu F., Xu L., Zhao X., Li X. (2020). The suitability of NONO-TFE3 dual-fusion FISH assay as a diagnostic tool for NONO-TFE3 renal cell carcinoma. Sci. Rep..

[31] Gupta S., Argani P., Jungbluth A.A., Chen Y.B., Tickoo S.K., Fine S.W., Gopalan A., Al-Ahmadie H.A., Sirintrapun S.J., Sanchez A. (2019). TFEB Expression Profiling in Renal Cell Carcinomas: Clinicopathologic Correlations. Am. J. Surg. Pathol..

[32] Xia Q.Y., Wang X.T., Fang R., Wang Z., Zhao M., Chen H., Chen N., Teng X.D., Wang X., Wei X. (2020). Clinicopathologic and Molecular Analysis of the TFEB Fusion Variant Reveals New Members of TFEB Translocation Renal Cell Carcinomas (RCCs): Expanding the Genomic Spectrum. Am. J. Surg. Pathol..

[33] Antic T., Taxy J.B., Alikhan M., Segal J. (2017). Melanotic Translocation Renal Cell Carcinoma With a Novel ARID1B-TFE3 Gene Fusion. Am. J. Surg. Pathol..

[34] Huang W., Goldfischer M., Babyeva S., Mao Y., Volyanskyy K., Dimitrova N., Fallon J.T., Zhong M. (2015). Identification of a novel PARP14-TFE3 gene fusion from 10-year-old FFPE tissue by RNA-seq. Genes Chromosomes Cancer.

[35] Malouf G.G., Su X., Yao H., Gao J., Xiong L., He Q., Comperat E., Couturier J., Molinie V., Escudier B. (2014). Next-generation sequencing of translocation renal cell carcinoma reveals novel RNA splicing partners and frequent mutations of chromatin-remodeling genes. Clin. Cancer Res..

[36] Wei S., Testa J.R., Argani P. (2022). A review of neoplasms with MITF/MiT family translocations. Histol. Histopathol..

[37] Garraway L.A., Widlund H.R., Rubin M.A., Getz G., Berger A.J., Ramaswamy S., Beroukhim R., Milner D.A., Granter S.R., Du J. (2005). Integrative genomic analyses identify MITF as a lineage survival oncogene amplified in malignant melanoma. Nature.

[38] Cronin J.C., Wunderlich J., Loftus S.K., Prickett T.D., Wei X., Ridd K., Vemula S., Burrell A.S., Agrawal N.S., Lin J.C. (2009). Frequent mutations in the MITF pathway in melanoma. Pigment Cell Melanoma Res..

[39] Yokoyama S., Woods S.L., Boyle G.M., Aoude L.G., MacGregor S., Zismann V., Gartside M., Cust A.E., Haq R., Harland M. (2011). A novel recurrent mutation in MITF predisposes to familial and sporadic melanoma. Nature.

[40] Bertolotto C., Lesueur F., Giuliano S., Strub T., de Lichy M., Bille K., Dessen P., d’Hayer B., Mohamdi H., Remenieras A. (2011). A SUMOylation-defective MITF germline mutation predisposes to melanoma and renal carcinoma. Nature.

[41] Nguyen K.A., Syed J.S., Espenschied C.R., LaDuca H., Bhagat A.M., Suarez-Sarmiento A., O’Rourke T.K., Brierley K.L., Hofstatter E.W., Shuch B. (2017). Advances in the diagnosis of hereditary kidney cancer: Initial results of a multigene panel test. Cancer.

[42] Lang M., Vocke C.D., Ricketts C.J., Metwalli A.R., Ball M.W., Schmidt L.S., Linehan W.M. (2021). Clinical and Molecular Characterization of Microphthalmia-associated Transcription Factor (MITF)-related Renal Cell Carcinoma. Urology.

[43] Miller A.J., Levy C., Davis I.J., Razin E., Fisher D.E. (2005). Sumoylation of MITF and its related family members TFE3 and TFEB. J. Biol. Chem..

[44] Murakami H., Arnheiter H. (2005). Sumoylation modulates transcriptional activity of MITF in a promoter-specific manner. Pigment. Cell Res..

[45] Vertegaal A.C.O. (2022). Signalling mechanisms and cellular functions of SUMO. Nat. Rev. Mol. Cell Biol..

[46] Rosonina E. (2019). A conserved role for transcription factor sumoylation in binding-site selection. Curr. Genet..

[47] Bonet C., Luciani F., Ottavi J.F., Leclerc J., Jouenne F.M., Boncompagni M., Bille K., Hofman V., Bossis G., Marco de Donatis G. (2017). Deciphering the Role of Oncogenic MITFE318K in Senescence Delay and Melanoma Progression. J. Natl. Cancer Inst..

[48] Schmidt L.S., Linehan W.M. (2016). Genetic predisposition to kidney cancer. Semin. Oncol..

[49] Hong S.B., Oh H., Valera V.A., Baba M., Schmidt L.S., Linehan W.M. (2010). Inactivation of the FLCN tumor suppressor gene induces TFE3 transcriptional activity by increasing its nuclear localization. PLoS ONE.

[50] Alesi N., Akl E.W., Khabibullin D., Liu H.J., Nidhiry A.S., Garner E.R., Filippakis H., Lam H.C., Shi W., Viswanathan S.R. (2021). TSC2 regulates lysosome biogenesis via a non-canonical RAGC and TFEB-dependent mechanism. Nat. Commun..

